# Characterization of the IGF2 Imprinted Gene Methylation Status in Bovine Oocytes during Folliculogenesis

**DOI:** 10.1371/journal.pone.0142072

**Published:** 2015-10-30

**Authors:** Anelise dos Santos Mendonça, Ana Luíza Silva Guimarães, Naiara Milagres Augusto da Silva, Alexandre Rodrigues Caetano, Margot Alves Nunes Dode, Maurício Machaim Franco

**Affiliations:** 1 Laboratory of Animal Reproduction, Embrapa Genetic Resources and Biotechnology, Brasília, Distrito Federal, Brazil; 2 Institute of Genetics and Biochemistry, Federal University of Uberlândia, Uberlândia, Minas Gerais, Brazil; 3 School of Agriculture and Veterinary Medicine, University of Brasília, Brasília, Distrito Federal, Brazil; 4 Embrapa Genetic Resources and Biotechnology, Brasília, Distrito Federal, Brazil; 5 School of Veterinary Medicine, Federal University of Uberlândia, Uberlândia, Minas Gerais, Brazil; Qingdao Agricultural University, CHINA

## Abstract

DNA methylation reprogramming occurs during mammalian gametogenesis and embryogenesis. Sex-specific DNA methylation patterns at specific CpG islands controlling imprinted genes are acquired during this window of development. Characterization of the DNA methylation dynamics of imprinted genes acquired by oocytes during folliculogenesis is essential for understanding the physiological and genetic aspects of female gametogenesis and to determine the parameters for oocyte competence. This knowledge can be used to improve *in vitro* embryo production (IVP), specifically because oocyte competence is one of the most important aspects determining the success of IVP. Imprinted genes, such as IGF2, play important roles in embryo development, placentation and fetal growth. The aim of this study was to characterize the DNA methylation profile of the CpG island located in IGF2 exon 10 in oocytes during bovine folliculogenesis. The methylation percentages in oocytes from primordial follicles, final secondary follicles, small antral follicles, large antral follicles, MII oocytes and spermatozoa were 73.74 ± 2.88%, 58.70 ± 7.46%, 56.00 ± 5.58%, 65.77 ± 5.10%, 56.35 ± 7.45% and 96.04 ± 0.78%, respectively. Oocytes from primordial follicles showed fewer hypomethylated alleles (15.5%) than MII oocytes (34.6%) (*p* = 0.039); spermatozoa showed only hypermethylated alleles. Moreover, MII oocytes were less methylated than spermatozoa (*p*<0.001). Our results showed that the methylation pattern of this region behaves differently between mature oocytes and spermatozoa. However, while this region has a classical imprinted pattern in spermatozoa that is fully methylated, it was variable in mature oocytes, showing hypermethylated and hypomethylated alleles. Furthermore, our results suggest that this CpG island may have received precocious reprogramming, considering that the hypermethylated pattern was already found in growing oocytes from primordial follicles. These results may contribute to our understanding of the reprogramming of imprinted genes during bovine oogenesis.

## Introduction

DNA methylation, an epigenetic event, regulates important biological processes, such as genomic imprinting, transposon silencing and chromosomal stability, and has an essential role in mammalian gametogenesis and embryogenesis [1–4].

During the mammalian life cycle, two waves of DNA methylation reprogramming occur. The first one takes place during gametogenesis, where primordial germ cells (PGCs) are demethylated to allow them to acquire a specific pattern of methylation according to the individual’s sex [3–5]. The second wave of methylation reprogramming starts immediately after fertilization. Paternal and maternal genomes are actively and passively demethylated, respectively, leading embryonic cells to a pluripotent state. Then, a *de novo* methylation process is initiated, specifically at the 8–16 cell and blastocyst stage in bovine and mouse embryos, respectively [6]. From this point, embryonic cells start receiving tissue-specific methylation patterns [3–5]. Therefore, a wide epigenetic reprogramming, which includes DNA methylation, post-translational histone modifications and other molecular events, is requisite for the production of viable gametes and embryos. Thus, understanding the life cycle of DNA methylation that occurs during gametogenesis may contribute to improving fertility traits in animals and increasing the efficiency of assisted reproductive technologies (ARTs), especially because epigenetic events may be susceptible to environment effects [7].

DNA methylation is involved in chromatin remodeling at imprinted and non-imprinted regions of the genome. At imprinted regions, DNA methylation patterns are acquired in a sex-specific manner during oogenesis and spermatogenesis [5]. These genomic regions are responsible for the regulation of imprinted genes, which have mono-allelic expression according to the parental origin [8–10]. Usually, imprinted genes are organized in clusters in the genome and are involved with embryo development, X-chromosome inactivation, placentation, fetal growth, etc [7, 11]. Accordingly, in this study, we chose to characterize the DNA methylation programming in a DMR that is involved in controlling an important imprinted gene, insulin-like growth factor 2 (IGF2). IGF2 is related to fetal growth [12], placenta development and tissue differentiation [13], and as an imprinted gene, is paternally expressed [13], being controlled by three intragenic DMRs and one intergenic DMR [7, 13–16]. Our laboratory previously characterized the methylation pattern of the intragenic CpG island located in exon 10 of IGF2 during the *in vitro* maturation of bovine oocytes [17], but we still need to characterize this pattern of methylation throughout oogenesis.

An essential epigenetic event that occurs during the initial embryonic development in female mammals is the X chromosome inactivation (XCI) [18–20], which is determinant to the viability of female embryos. One of the most important genes related to the initiation of the XCI is the XIST gene [21–23], which has its expression controlled by DNA methylation. In this study, we chose to analyse the profile of methylation of XIST as an unmethylated control.

Oocyte quality is one of the most important aspects related to the efficiency of embryo production and fertility in animals [24]. A cycle of epigenetic reprogramming occurs during oogenesis [25], and correct DNA methylation reprogramming is directly correlated to oocyte quality [26]. Therefore, understanding this reprogramming of imprinted genes during oogenesis is essential to support the improvement of fertility and *in vitro* embryo production in animals and humans.

The aim of this study was to characterize the DNA methylation profile of the CpG island located in exon 10 of the IGF2 gene in oocytes during bovine folliculogenesis.

## Material and Methods

The Ethics Committee of the Federal University of Uberlândia–CEUA/UFU–Uberlândia, MG, Brazil approved this experiment (007/12).

### Oocyte recovery, classification and *in vitro* maturation

Ovaries from crossbred cows (*Bos taurus indicus x Bos taurus taurus*), that are aged between 30 and 72 months, were collected immediately after slaughter at a local abattoir (Qualimaxima, Luziânia, Goiás, Brazil). They were immediately transported to the laboratory in saline solution (0.9% NaCl) supplemented with penicillin (100 IU/ml) and streptomycin (100 mg/ml; (Sigma, St. Louis MO, USA) at 35–37°C.

Cumulus oocyte complexes (COCs) were recovered and classified according to procedures previously established in our laboratory [27, 28]. Briefly, the ovarian cortex was separated with a scalpel blade and cut longitudinally, transversally and obliquely with a *Tissue Chopper* (The Mickle Laboratory Engineering Co. Ltd., Gomshall, Surrey, England). The cuts were 150, 200, 250, 300 and 350 μm thick. The entire process was performed using phosphate-buffered saline (PBS) containing 10% fetal calf serum (FCS; (Gibco BRL, Burlington, ON, Canada). The ovarian fragments were placed in 50 mL conical tubes along with approximately 5 mL of PBS supplemented with 10% fetal calf serum (FCS). A 3 mL Pasteur pipette was used to mechanically dissociate the oocytes with successive suspension (10 to 40 times). The resulting material was filtered using 500 and 245 μm nylon mesh to the collect large and small oocytes, respectively. After decantation, 1 mL of the pellet was analyzed using an inverted microscope (Axiovert 135 M, Zeiss, Germany).

Oocytes with homogeneous cytoplasm and free of granulosa cells (denuded by pipetting) were transferred to a 10 mL drop of tissue culture medium-199 (TCM-199) supplemented with Hank's Balanced Salt (Gibco BRL, Burlington, ON, Canada). After many washes to remove any granulosa cells and impurities, the isolated oocytes were photographed and measured using the Motic Images Plus 2.0 program (Motic China Group Co. Ltd., Xiamen, China). The diameter measurement was performed by excluding the zona pellucida. Oocytes < 20 μm, 65–85 μm, 100–120 μm (from 1–3 mm follicles) and >128 μm (from ≥ 6 mm follicles) in diameter, were classified as oocytes from primordial follicles, final secondary follicles, small antral follicles and large antral follicles, respectively. Oocytes from primordial and final secondary follicles represent the preantral phase of folliculogenesis [27]. The antral groups were selected according to a study that was performed in our laboratory, which showed that oocytes from 1–3 mm follicles are less competent for embryo production than the oocytes from follicles ≥6 mm in size [28]. Oocytes were washed four times with PBS without calcium or magnesium. Then, the oocytes were stored at -80°C until DNA isolation.

For oocyte maturation, COCs from 3–8 mm in diameter follicles, which are routinely used for IVP, were aspirated. Only COCs with homogeneous granulated cytoplasm and at least three layers of compact cumulus cells were used. After selection, COCs were washed and transferred to 50 ml (≤10 oocytes) of maturation medium, covered with silicone oil and incubated for 22–24 h at 39°C at 5% CO_2_. The maturation medium consisted of TCM-199 (Invitrogen, CA, USA) supplemented with 10% FCS (Gibco BRL, Burlington, ON, Canada), 12 IU/ml LH (Sigma, St. Louis MO, USA), 0.1 IU/ml FSH (Sigma, St. Louis MO, USA), 0.1 mg/ml L-glutamine (Sigma, St. Louis MO, USA) and antibiotic (amikacin, 0.075 mg/ml). Following the maturation period, COCs were incubated with 0.2% hyaluronidase for 10 min and then denuded by repeated pipetting. Only oocytes that had extruded their first polar body were considered matured (mature MII oocytes) and used for DNA isolation.

### Sperm processing

Sperm DNA from a sexually mature Nellore (*Bos taurus indicus*) bull of proven fertility and routinely used for IVP in our laboratory was used as a control for the DNA methylation patterns. These sperm cells were prepared as described by Carvalho et al. [29].

### DNA isolation and sodium bisulfite treatment

Two pools of 70 oocytes per group of immature oocytes (primordial, final secondary, small antral and large antral), one pool of 63 MII oocytes and three straws of semen were used for DNA isolation and bisulfite treatment. Each pool of oocytes was treated with Pronase E (Sigma, St. Louis MO, USA) to digest the zona pellucida at a final concentration of 10 mg/mL. Next, the oocytes were incubated in a thermocycler (PXE 0.5 Thermal Cycler, Electron Corporation, Asheville, NC, USA) for 35 minutes at 37°C followed by 15 minutes at 85°C. The genomic DNA was extracted by cellular lysis using heat shock, in which the samples were frozen in liquid nitrogen and immediately placed in a thermocycler for 1 minute at 95°C. This procedure was repeated four times. Genomic DNA from sperm was isolated from pellets obtained after passage through a Percoll gradient using the salting out procedure as described in Carvalho et al. [29].

The DNA samples were treated with sodium bisulfite using the EZ DNA Methylation kit^®^ (Zymo Research, Irvine, CA, USA) according to manufacturer’s protocol. The samples were diluted with 12 μL of distilled water and stored at -80°C until PCR amplification.

### PCR amplification, cloning, and bisulfite sequencing

**Fig 1:**
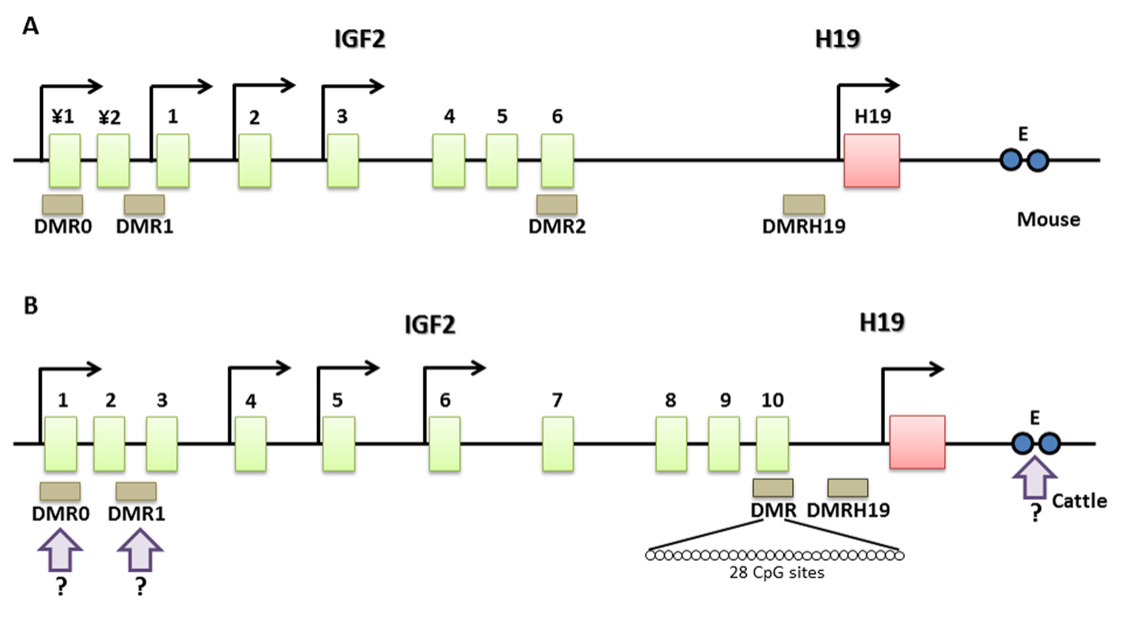
Comparative representation of the mouse and cattle IGF2 genes. Green rectangles represent exons, while pink rectangles represent the H19 gene. Promoter regions are indicated by arrows and differentially methylated regions (DMRs) are represented by brown rectangles. ¥1 and ¥2 represent pseudo-exons 1 and 2, respectively, in the mouse igf2 gene. The CpG island analyzed in this study is located in IGF2 exon 10. White circles represent each individual CpG that was analyzed. The enhancer (E) that is involved in controlling the H19 and igf2 genes is represented by two blue circles. Question marks means that the information has not been completely confirmed in cattle.

Sodium bisulfite-treated DNA samples were subjected to nested PCR. The primer sequences, *GenBank* accession number, CpG island position and amplicon size are listed in Table 1. The molecular structure of the bovine IGF2 gene showing the CpG island that was analyzed in this study is illustrated in Fig 1.

**Table 1:** Gene identification, primer sequence and annealing position, *GenBank* accession number, CpG island position and amplicon size.

Gene	Primers sequences (5’–3’)	Primer annealing position	*GenBank* accession	CpG island position	*Amplicon* size
IGF2*out	F: TGGGTAAGTTTTTTTAATATGATATT	243–268	X53553.1	Exon 10	455 bp
	R: TTTAAAACCAATTAATTTTATACATT	672–697			
IGF2*inner	F: TAATATGATATTTGGAAGTAGT	257–278	X53553.1	Exon 10	420 bp
	R: ACATTTTTAAAAATATTATTCT	655–676			
XIST** out	F: GGGTGTTTTTGTTTTAGTGTGTAGTA	1127–1252	AJ421481.1	Exon 1	482 bp
	R: CTTTAATACCACCCACTAAAATTAATAC	1581–1608			
XIST** inner	F: TTGTTATATAGTAAAAGATGGT	1169–1190	AJ421481.1	Exon 1	405 bp
	R: ACCAATCCTAACTAACTAAATA	1552–1573			

*Gebert et al. [13]

**Liu et al. [30]. F-forward; R-Reverse; bp-base pair

The two rounds of amplification for IGF2 and x-inactive specific transcript (XIST), used as unmethylayed control, were performed in a total volume of 20 μL using 1X Taq buffer, 2.0 mM MgCl_2_, 0.4 mM dNTPs, 1 U Platinum^®^ Taq polymerase (Invitrogen, CA, USA), 1 μM of each primer (forward and reverse) and 3 μL of bisulfite-treated DNA for the first round and 0.5 μL of the amplicon for the second round. The temperature and time conditions for each PCR are presented in Table 2.

**Table 2:** Nested PCR conditions for the IGF2 and XIST genes.

Gene	Reaction	Initial Denaturing	Cycles (45 and 40 cycles for each reaction for IGF2 and XIST, respectively)			Final Extension
			Denaturing	Annealing	Extension	
**IGF2**	1^a^ reaction	94°C; 3 min.	94°C; 40 s.	45°C; 1 min.	72°C; 1 min.	72°C; 15 min.
	2^a^ reaction	94°C; 3 min.	94°C; 40 s.	40°C; 1 min.	72°C; 1 min.	72°C; 15 min.
**XIST**	1^a^ reaction	94°C; 7 min.	94°C; 45 s.	47°C; 1 min. and 30 s.	72°C; 1 min.	72°C; 15 min.
	2^a^ reaction	94°C; 4 min.	94°C; 40 s.	42°C; 45 s.	72°C; 45 s.	72°C; 15 min.

After the nested PCR, the amplicons were purified from an agarose gel using the Wizard^®^ SV Gel and PCR Clean-Up System (Promega, Madison, WI, USA) according to the manufacturer’s protocol. Then, the purified amplicons were cloned into the TOPO TA Cloning^®^ vector (Invitrogen, CA, USA) and transferred into DH5α cells using a heat shock protocol. Plasmid DNA was isolated using the QIAprep Spin Miniprep Kit (Qiagen, CA, USA) and individual clones were sequenced using the dideoxy methodology. The sequencing quality was analyzed using Chromas^®^ and the methylation pattern was analyzed using the BiQ Analyser^®^ program [31]. DNA sequences were compared with GenBank X53553.1 for IGF2. Only sequences originating from the clones with ≥ 95% homology and cytosine conversion were used.

### Statistical analysis

The methylation pattern data were compared among experimental groups using ANOVA and Tukey’s test or the Kruskal-Wallis and Mann-Whitney tests for data showing normality or not, respectively. The frequency of hypomethylated and hypermethylated (more than 50% of methylated CpGs in a sequence, determined according to the method of Imamura et al. [32]) alleles were compared using the χ^2^ test. All of the analyses were performed using Systat, version 10.2 (Inc., Richmond, CA, USA) and the results are presented as the mean ± standard error of the mean (SEM).

## Results

**Fig 2:**
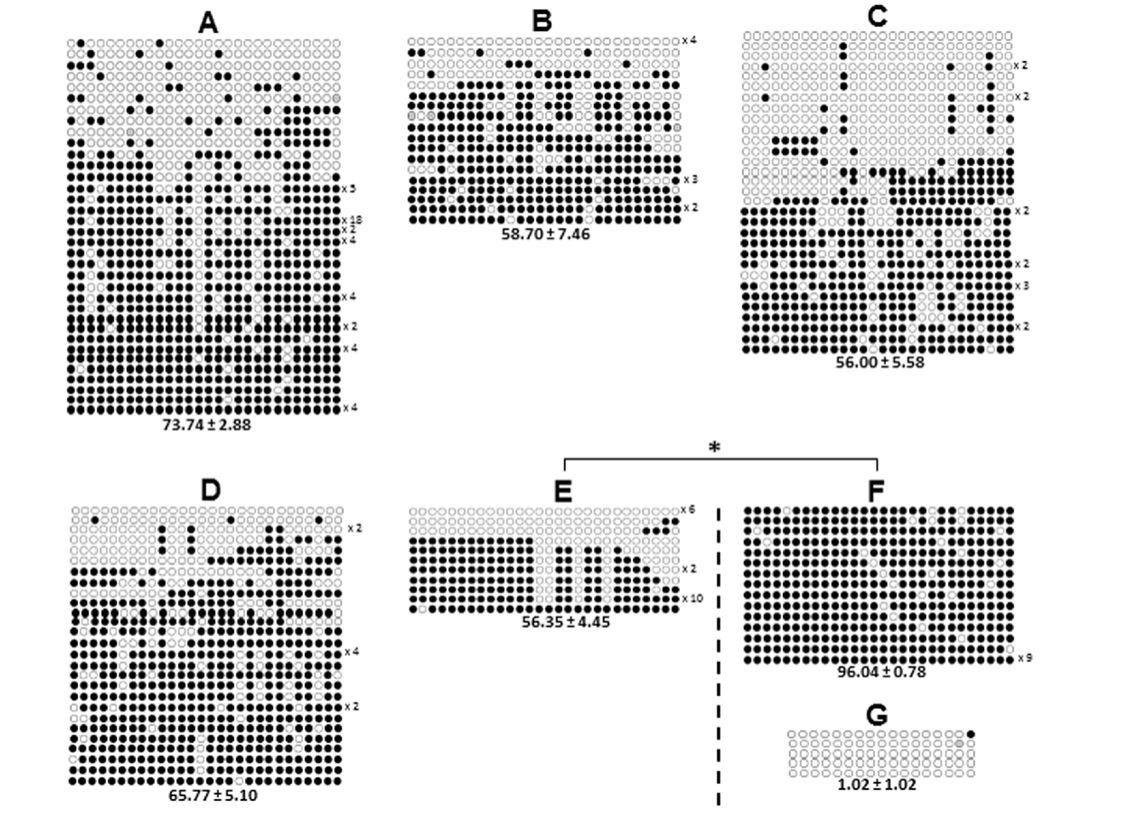
Dynamics of DNA methylation on the last exon of the IGF2 gene from oocytes during bovine folliculogenesis. Letters A-E represent the oocyte groups and F and G spermatozoa. (A) Oocytes from primordial follicles; (B) oocytes from final secondary follicles; (C) oocytes from small antral follicles; (D) oocytes from large antral follicles; (E) matured MII oocytes; (F and G) matured spermatozoa. A-F show the methylation profile on the last exon of IGF2 and G shows the methylation profile on the exon 1 of the XIST. Each line represents one individual clone and each circle represents one CpG dinucleotide (28 CpGs for IGF2 and 17 CpGs for XIST). White circles represent unmethylated CpGs, filled black circles represent methylated CpGs and gray circles represent a CpG that could not be analyzed. The numbers to the right of each clone indicate the number of times that the allele was sequenced and the numbers to the bottom of each group represent the DNA methylation means ± standard errors for each group. (*) represent significantly different means (*p* ≤ 0.05). XIST was used as unmethylated control.

**Fig 3:**
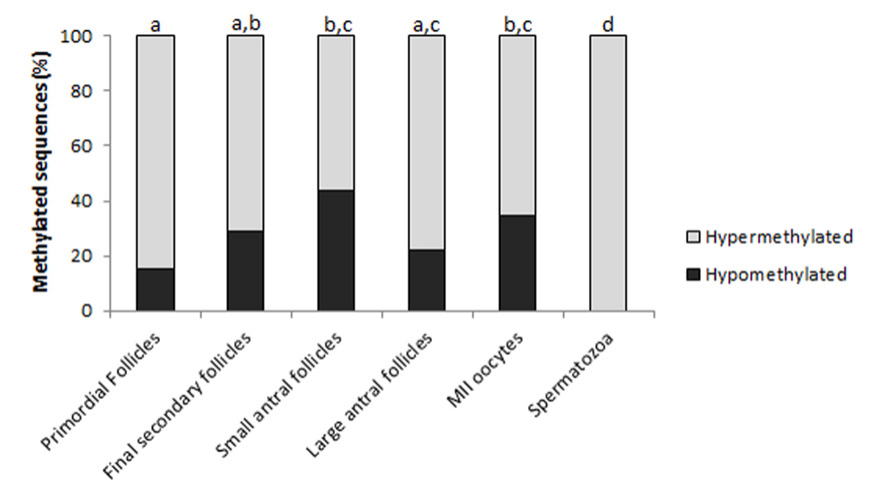
Frequency of IGF2 hypermethylated and hypomethylated alleles. Each bar represents a treatment group that was analyzed (oocytes from primordial, final secondary, small antral and large antral follicles, MII oocytes and spermatozoa). The black part of the bar represents the frequency or proportion of hypomethylated alleles and gray represents the hypermethylated alleles. Different letters represent significant differences between the groups according to the χ^2^ test (*p* ≤ 0.05).

The methylation profile of the IGF2 gene is shown in Fig 2. The methylation percentage, number of analyzed sequences, minimum number of alleles according to the methylation pattern and number of hypermethylated sequences are shown in Table 3. Fig 3 shows the frequency between the hypermethylated and hypomethylated alleles for the IGF2 gene.

**Table 3:** The methylation percentage, number of analyzed sequences, minimum number of alleles and number of hypermethylated sequences (greater than 50% of methylated CpG sites) for each oocyte group and spermatozoa for the IGF2 gene.

Follicle category	DNA methylation percentage ± SEM	Number of analyzed sequences	Minimum number of alleles	Number of hypermethylated sequences
**Primordial**	73.74 ± 2.88^a^	71	35	60 (84.5%)^a^
**Final Secondary**	58.70 ± 7.46^a^ ^,^ ^b^	24	18	17 (70.8%)^a^ ^,^ ^b^
**Small Antral**	56.00 ± 5.58^b^	39	31	22 (56.4%)^b^ ^,^ ^c^
**Large Antral**	65.35 ± 7.45^a^ ^,^ ^b^	32	27	25 (78.1%)^a^ ^,^ ^c^
**MII oocytes**	56.35 ± 7.45^a^ ^,^ ^b^	26	11	17 (65.4%)^b^ ^,^ ^c^
**Spermatozoa**	96.04 ± 0.78^c^	23	15	23 (100%)^d^

^a, b, c, d^ Different letters indicate significant differences among groups within each gene; *p* ≤ 0.05

No significant differences in DNA methylation percentage were found between the oocyte from primordial follicles and MII oocytes (*p* = 0.088). *In vitro* matured oocytes (MII) were less methylated than mature spermatozoa (*p*<0.001) (Fig 2). Regarding the frequency of hypermethylated and hypomethylated IGF2 alleles, oocytes from primordial follicles showed fewer hypomethylated alleles than oocytes from small antral follicles (*p* = 0.001) and MII oocytes (*p* = 0.039); spermatozoa showed only hypermethylated alleles (Fig 3).

## Discussion

To characterize the DNA methylation pattern during bovine folliculogenesis for the imprinted gene IGF2, we used oocytes from follicles representing the initial and final phases of the two stages of folliculogenesis, the preantral and antral stages. Thus, we have collected oocytes from primordial, final secondary, small antral and large antral follicles using methodologies that were previously established in our laboratory [17, 27, 28].

Our laboratory is interested in evaluating the influence of assisted reproductive technologies (ARTs) on the DNA methylation patterns of imprinted genes involved in bovine embryo development. Thus, we are investigating the influence of ARTs on the DNA methylation pattern of the IGF2 gene, specifically in the CpG island located in exon 10 of the gene [7, 17, 29, 33].

In this study, we have characterized the methylation pattern of this CpG island from oocytes throughout bovine folliculogenesis. The comparative gene structure of IGF2 showing all of the DMRs involved in mouse and bovine IGF2 expression, including the CpG island examined in this study, is illustrated in Fig 1. Despite the mechanism controlling IGF2 expression is not well characterized in cattle as in mouse, it is known that bovine IGF2 shows paternal mono-allelic expression [34].

We found that the CpG island shows a similar methylation pattern in all of the oocyte groups. Fully-grown MII oocytes showed 56.35% methylation (Fig 2) with ∼35% hypomethylated alleles (Fig 3). In contrast, mature spermatozoa showed 96% methylation with 100% hypermethylated alleles (Figs 2 and 3). Even though this region does not show a classical DMR pattern with one allele totally methylated and the other totally demethylated, the methylation patterns of the MII oocytes and spermatozoa were significantly different (Figs 2 and 3 and Table 3). These observations are supported by data from Gebert et al. and Fagundes et al. [13, 17] that found 16% and 28% methylation, respectively, for this CpG island, and conclude that this genomic region is a DMR [13, 17]. The discordance among these data and our findings may be related to differences in the *in vitro* culture conditions/oocyte donors (*Bos taurus taurus* x *Bos taurus indicus*) and the sizes of the collected oocytes in the Gebert et al. and Fagundes et al. studies, respectively [13, 17]. In contrast, when analyzing the same immature oocyte groups, from follicles with 1–3 mm in size and without any influence of *in vitro* culture, we have found the same methylation pattern as our previous study [17], with 56.0% and 51.1% of methylation, respectively, which reinforces the agreement of these studies. Taken together, it may be suggested that this CpG island may be a gametic or primary DMR, as described by Colosimo et al. [35] and Yuen et al. [36], and that is imprinted in bovine.

In agreement with data from Gebert et al. and Fagundes et al. [13, 17], we also observed the presence of both hypomethylated and hypermethylated alleles in all of the oocyte groups except spermatozoa (Fig 2 and Table 3). These findings suggest that the methylation pattern for this CpG island changes from a hypermethylated to a hypomethylated state during bovine folliculogenesis, where oocytes from primordial follicles showed a fewer number of hypomethylated alleles compared to MII oocytes (*p* = 0.039) (Fig 3). This also supports the results from Gebert et al. [13] and a previous study by our group [17] that found a hypomethylated profile in fully-grown MII oocytes. The hypermethylated pattern found in growing oocytes from primordial follicles (Figs 2 and 3 and Table 3) may indicate precocious reprogramming, indicative of species-specific differences as suggested by Colosimo et al. [35]. Taken together, these results suggest that this CpG island is still reprogramming during bovine oocyte *in vitro* maturation, resulting in a hypomethylated state in matured MII oocytes compared to that in sperm.

O’Doherty, O’Shea and Fair [37] evaluated the methylation pattern of imprinted genes in bovine growing oocytes during the antral phase of folliculogenesis. For the majority of the genes evaluated, the methylation pattern increased substantially during oocyte growth [37], in agreement with what is expected for the imprinted genes as their DNA methylation pattern is established during the growth stage [38]. Nonetheless, some of the genes did not show substantial changes in their methylation profiles during oocyte growth, which is similar to what we found for IGF2 in this study. These results are evidence that imprinted genes are not reprogrammed in the same manner and at the same time during oogenesis, even in the same species.

The results obtained in this study can contribute to improving our understanding of methylation reprogramming of imprinted genes during oogenesis in cattle. It is important to analyze the methylation pattern of the genomic region examined here in *in vivo*-matured bovine oocytes to completely understand methylation reprogramming in bovine folliculogenesis oogenesis, verify the influence of the *in vitro* maturation process on epigenetic features and, consequently, enhance oocyte quality and the efficiency of *in vitro* embryo production.
